# Mutualistic interaction between *Salmonella enterica* and *Aspergillus niger* and its effects on *Zea mays* colonization

**DOI:** 10.1111/1751-7915.12182

**Published:** 2014-10-29

**Authors:** Roberto Balbontín, Hera Vlamakis, Roberto Kolter

**Affiliations:** Department of Microbiology and Immunobiology, Harvard Medical School77 Avenue Louis Pasteur, HIM building, Room #1042, Boston, MA, 02115, USA

## Abstract

*Salmonella* Typhimurium inhabits a variety of environments and is able to infect a broad range of hosts. Throughout its life cycle, some hosts can act as intermediates in the path to the infection of others. *Aspergillus niger* is a ubiquitous fungus that can often be found in soil or associated to plants and microbial consortia. Recently, *S*. Typhimurium was shown to establish biofilms on the hyphae of *A. niger*. In this work, we have found that this interaction is stable for weeks without a noticeable negative effect on either organism. Indeed, bacterial growth is promoted upon the establishment of the interaction. Moreover, bacterial biofilms protect the fungus from external insults such as the effects of the anti-fungal agent cycloheximide. Thus, the *Salmonella–Aspergillus* interaction can be defined as mutualistic. A tripartite gnotobiotic system involving the bacterium, the fungus and a plant revealed that co-colonization has a greater negative effect on plant growth than colonization by either organism in dividually. Strikingly, co-colonization also causes a reduction in plant invasion by *S*. Typhimurium. This work demonstrates that *S*. Typhimurium and *A. niger* establish a mutualistic interaction that alters bacterial colonization of plants and affects plant physiology.

## Introduction

*Salmonella enterica* serovar Typhimurium (*S.* Typhimurium hereafter) and *Aspergillus niger* are two important model systems in the study of microbial pathogens. *Aspergillus niger* is distributed worldwide and can colonize diverse habitats and hosts (Wilson *et al*., 2002; Nielsen *et al*., 2009). *Aspergillus* species are successful symptomless endophytes and pre- and post-harvest pathogens of plants (Perrone *et al*., 2007; Palencia *et al*., 2010). *Salmonella* Typhimurium survives in different environments and is able to colonize a plethora of hosts, causing from no symptoms to death (Baumler *et al*., 1998). The life cycle of *S.* Typhimurium comprises an infection/persistence phase within the host and a survival/spread stage in the external environment while transitioning to a new host (Foltz, 2009; Thomason *et al*., 1977). Plants play a key role in the survival and dissemination of *S.* Typhimurium in the environment. Indeed, *Salmonella* outbreaks have been often linked to the consumption of foods of plant origin (Brandl *et al*., 2013). In both phases, *S.* Typhimurium interacts with a number of other microorganisms. These interactions can be synergistic, neutral or antagonistic, and might influence the colonization of a given niche/host by this bacterium. For example, several members of the intestinal flora have an antagonistic effect on gut colonization by *S.* Typhimurium (Servin, 2004). Moreover, antagonism by other microbes can occur outside of the host as well (Servin, 2004). In contrast, gut inflammation induced by *S.* Typhimurium causes changes in the composition of the intestinal microbiota (Thiennimitr *et al*., 2012). Furthermore, when co-infection with *Plasmodium* species occurs, the ability of *S.* Typhimurium to cause systemic infection in humans increases (Roux *et al*., 2010). Finally, plant infection by *S.* Typhimurium is also facilitated by the presence of other pathogens (Meng *et al*., 2013; Potnis *et al*., 2014).

Bacteria and fungi are often found associated in nature, in soils or in association with plants and animals. Fungal–bacterial interactions can positively or negatively affect either participant (Wargo and Hogan, 2006). For instance, the interaction between *A. niger* and *Collimonas fungivorans* results in bacterial mycophagia (Mela *et al*., 2011) and *S.* Typhimurium can kill the fungal pathogen *Candida albicans* (Tampakakis *et al*., 2009; Kim and Mylonakis, 2011). In contrast, the co-incubation of *A. niger* with *Bacillus subtilis* leads to a metabolic change and downregulation of defence mechanisms in both microbes, suggesting a neutral interaction (Benoit *et al*., 2014). Alternatively, members of the *Aspergillus* genus interact beneficially with mycobacteria by facilitating bacterial infection (Mussaffi *et al*., 2005).

A recent study reported that *S.* Typhimurium forms biofilms on the hyphae of *A. niger*, and found that the association depends on the interaction between bacterial cellulose and fungal chitin (Brandl *et al*., 2011). Curli fibres, which are important for biofilm formation on inert surfaces (reviewed in Barnhart and Chapman, 2006), were important for biofilm maintenance on *A. niger* hyphae but not necessary for early attachment (Brandl *et al*., 2011). Mutants in *csgD*, which do not produce curli, form biofilms on *A. niger* that breakdown by 7 h of colonization and are almost completely detached by 24 h (Brandl *et al*., 2011). Importantly, the biological consequences of the relationship between *A. niger* and *S.* Typhimurium remained unexplored.

Here, we provide evidence that the interaction between *S.* Typhimurium and *A. niger* is mutualistic. Moreover, our results demonstrate that the fungal–bacterial interaction modifies the effects of the microbes on maize plants. In addition, we found that *S.* Typhimurium can invade maize plants and internalization is affected by the interaction with *A. niger*.

## Experimental procedures

### Strains, media and culture conditions

Strains used in this work are listed in Table S1. All *Salmonella enterica* strains belong to the serovar Typhimurium strain ATCC 14028 (Jarvik *et al*., 2010). *Salmonella* Typhimurium mutants were generated using the λ Red recombination system (Datsenko and Wanner, 2000; Murphy *et al*., 2000; Yu *et al*., 2000) and transferred to a clean background using P22 HT 105/1 *int201* transduction (Schmieger, 1972). To obtain phage-free isolates, transductants were purified by streaking on ‘green’ plates (Chan *et al*., 1972; Watanabe *et al*., 1972). Strain RB164 harbours a constitutively expressed superfolder green fluorescent protein (sfGFP) (Pédelacq *et al*., 2006) inserted in the chromosomal pseudogene locus *malX-malY* (Jarvik *et al*., 2010). RB164 was generated using isothermal assembly of polymerase chain reaction products (Gibson *et al*., 2009) and λ Red recombination. Primers used for the construction of this strain were ORB007, ORB002, ORB003 and ORB008 (Table S2). Template DNAs used were plasmids pXG-1 (Urban and Vogel, 2007) and pTB263 (Dinh and Bernhardt, 2011). Strains RB242, RB243 and RB244 are derivatives of strains SV6062, SV6063 and SV6106 (Baisón-Olmo *et al*., 2012), respectively, where the constitutive sfGFP from RB164 was introduced by transduction. Strains RB225, RB226 and RB229 derive from strains MA9999, MA10314 and MA8933 (Figueroa-Bossi *et al*., 2009), respectively, where the constitutive sfGFP from RB164 was introduced by transduction.

The *A. niger* strain used in this study (ZK3055) is a wild environmental isolate.

Solid Luria-Bertani (LB) medium contained agar at a 2% (w/v) final concentration. Antibiotics were used at the final concentrations described elsewhere (Maloy, 1990). All bacterial cultures were incubated in LB broth at 30°C and 130 r.p.m until late exponential growth. Bacterial cells were washed twice with 10 mM potassium phosphate buffer (pH 7) and resuspended in either 10 mM potassium phosphate buffer (pH 7) or 10% M9 (Miller, 1972) (v/v) supplemented with 0.01% sucrose (w/v).

*Aspergillus niger* was grown on potato dextrose agar at 20°C for 7 days. Spores were collected and stored in 0.2% Tween 80 (Sigma, St. Louis, MO, USA) (v/v) at 4°C and spore counts were determined with a haemocytometer. Potato dextrose broth was inoculated with 9 × 10^4^ spores ml^−1^ and incubated over night at 30°C and 130 r.p.m to promote spore germination. Then 5 ml of germinated spore suspension was added to 100 ml of M9 supplemented with 0.1% sucrose (w/v). The fungal culture was incubated at 30°C and 130 r.p.m for 24 h, and mycelia were washed five times with either 10 mM potassium phosphate buffer (pH 7) or 10% M9 (v/v) supplemented with 0.01% sucrose (w/v) prior to co-incubation with bacteria.

Co-incubations took place in either 10 mM potassium phosphate buffer (pH 7) or 10% M9 (v/v) supplemented with 0.01% sucrose (w/v), at 30°C and 130 r.p.m (unless indicated otherwise) for different periods of time prior to the corresponding analysis. Fungal concentration in co-cultures was 1 mycelial microcolony per millilitre and bacterial one was 2 × 10^7^ cells ml^−1^. In the experiments involving propidium iodide (PI; Figs 3 and 5), mycelia were incubated for 20 min in a freshly made solution at 2.5 μg ml^−1^ in 10 mM potassium phosphate buffer (pH 7), prior to the analysis. PI is red fluorescent when bound to nucleic acids and membrane impermeant. Therefore, PI is excluded from viable cells and only can penetrate cells when their membrane is compromised so it can be used to identify dead cells (Bjerknes, 1984).

### Microscopy

Samples were imaged with a Nikon Eclipse TE2000-U (Nikon, Tokyo, Japan) microscope equipped with a 20X Plan Apo (Nikon) objective. Pictures were taken with a Hamamatsu digital camera model ORCA-ER (Hamamatsu Photonics, Hamamatsu, Japan). Epifluorescence signal was detected using GFP (Chroma #41020) or Texas Red (Chroma #62002v2) filter sets. All images were taken at the same exposure time, processed identically for compared image sets, and prepared for presentation using MetaMorph (Molecular Devices, Sunnyvale, CA, USA) and ImageJ (public domain freeware) software. A minimum of three different positions for each of three independent biological replicates were analysed in microscopy experiments, and images shown in the figures are representative results. Fluorescence intensity of samples for Figs 3 and 5 was calculated using ImageJ software.

Alternatively, samples were imaged using a Zeiss Stemi SV6 stereoscope (Carl Zeiss Microscopy, Jena, Germany) attached to a fluorescence illumination system (X-cite 120, Lumen Dynamics; Excelitas Technologies Corp., Waltham, MA, USA). Pictures were taken with a Zeiss Color AxioCam (Carl Zeiss Microscopy). All images were taken at the same exposure time, processed identically for compared image sets and prepared for presentation using ImageJ software.

### Competition assays

Fungi (at 1 mycelia ml^−1^) were mixed with 2 × 10^7^ cells ml^−1^ of a 1:1 mixture of either ZK2851 (wild-type *S.* Typhimurium):RB231 (wild-type *S.* Typhimurium harbouring a tetracycline resistance gene inserted in a neutral chromosomal locus), ZK2851:RB206 (*cheY* mutant) or ZK2851:RB207 (*fliGHI* mutant) in 10 mM potassium phosphate buffer (pH 7) and incubated at 30°C without shaking for 1 h. Mycelia were then scooped out, washed three times with potassium phosphate buffer, sonicated (20 pulses of 1 s with 1 s interval, amplitude 30%, on a QSonica Q125 sonicator; Qsonica, Newtown, CT, USA) and vortexed for 10 s. These conditions were optimized to maximize bacterial detachment from the fungus as observed by microscopy while minimizing cell death. Then dilutions of bacterial solutions were plated onto selective media and colony-forming units (cfu) were calculated. Ratios of attached cells in the mutant versus the wild type were calculated and normalized to the ratio of the corresponding input mixture.

### Bacterial growth assays

Bacteria at 2 × 10^7^ cells ml^−1^ were incubated either alone, in the presence of live *A. niger* or in the presence of heat-killed (i.e. autoclaved) fungi (1 mycelia ml^−1^) in 24-well plates containing either 10 mM potassium phosphate buffer (pH 7) or 10% M9 (v/v) supplemented with 0.01% sucrose (w/v). At different time points, wells were sonicated (10 pulses of 1 s with 1 s interval, amplitude 30%), their contents were transferred to microcentrifuge tubes, sonicated again (10 pulses of 1 s with 1 s interval, amplitude 30%) and vortexed for 10 s. Then bacterial solutions were plated onto selective media and cfu were calculated and normalized to input values.

In experiments involving physical separation of fungi and bacteria (Fig. 4A), Millicell® Cell Culture Inserts (EMD Millipore, Merck KGaA, Darmstadt, Germany) were used. The membranes were reinforced by adding 100 μl of 0.7% agarose (w/v). Diffusion through the membrane was assessed by measuring optical density of coloured solutions.

### Plant experiments

*Zea mays* used in these experiments was the commercial variety Sugar Buns F1 (se+) obtained from Johnny Selected Seeds (Winslow, ME, USA). Seeds were surface sterilized with 70% ethanol (v/v) followed by 5% sodium hypochlorite (v/v) and rinsed three times with sterile distilled water. Seeds were incubated at 30°C in the dark for germination. Germinated seeds with roots of around 1 cm were planted on assay tubes containing 20 ml of Murashige-Skoog basal salt mixture (Sigma) at 4.3 g l^−1^ with 0.8% (w/v) agar and incubated in a growth chamber (24°C, 16 h daytime, 8 h dark time) for 4 days prior to inoculation. Plant roots were inoculated with 100 μl of 10 mM potassium phosphate buffer (pH 7) as a control or with buffer containing either 10^7^ cells of *S.* Typhimurium, 10^4^ spores of *A. niger* or a mixture of 10^7^ cells bacteria and 10^4^ fungal spores. Plants were then incubated in a growth chamber (24°C, 16 h daytime, 8 h dark time) for 14 days. Root or leaf samples were obtained and analysed. Root samples for fluorescence microscopy were obtained by peeling root epidermis using a sterile surgical blade and placing tissue fragments onto microscope slides. Leaf samples were obtained by cutting 1 cm of the tip of the flag leaf of each plant. The statistical analysis to evaluate the effect of the organisms on plants was carried out using one-way analysis of variance (ANOVA) (*P* < 0.01) on Gnumeric software (open-source public domain freeware).

## Results and discussion

### Bacterial attachment to fungi starts rapidly, is extensive, robust and does not occur on dead mycelia

**Fig 1:**
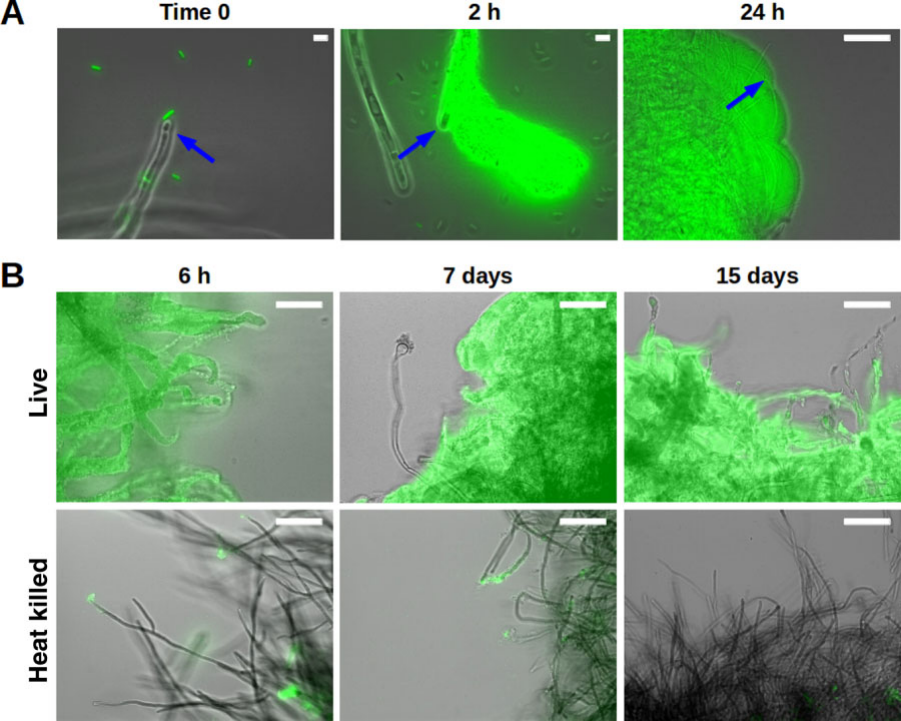
Bacteria require live fungus to form a biofilm. *In vitro* co-incubation of live and heat-killed mycelia of *A. niger* with sfGFP-labelled *S*. Typhimurium cells (false coloured green). Images are overlay of transmitted light (grey) with GFP fluorescence.A. Time-course of biofilm initiation on fungal hyphae (blue arrows) over a period of 24 h.B. Dense biofilms are formed on live hyphae (upper panels) after 6 h and are maintained for 15 days. In contrast, bacterial attachment to heat-killed mycelia (lower panels) is limited and disappears with time. Scale bars: 50 μm.

In order to study the *S.* Typhimurium–*A. niger* interaction over time, a co-culture system was developed. Diluted minimal medium supplemented with sucrose as the sole carbon source allowed for slow fungal growth and, because *S.* Typhimurium cannot metabolize sucrose (Gutnick *et al*., 1969), bacteria did not take over the culture. In order to facilitate visualization, the *S.* Typhimurium strain we used constitutively expressed sfGFP whereas the *A. niger* strain was not fluorescently labelled. Both microbes were co-cultured at 30°C with gentle shaking (130 r.p.m) and analysed by fluorescence microscopy at different time points (see *Experimental Procedures*). As has been previously reported (Brandl *et al*., 2011), at time zero (immediately after co-inoculation) we observed bacteria (false coloured green) approaching the tip of the hyphae (Fig. 1A, left panel). After 2 h, large bacterial aggregates were found at the extremes of the hyphae (Fig. 1A, centre panel). At 24 h, the fungus was completely covered by bacterial biofilms (Fig. 1A, right panel). In order to test the stability of the interaction and to determine if the fungus must be alive for bacteria to colonize it, we performed a longer assay in which fungal cells were either alive or heat killed prior to co-inoculation. Although the bacterial biofilm present on the live hyphae was stable and grew for over 2 weeks (Fig. 1B, upper panels), bacterial attachment to heat-killed mycelia was weak and disappeared over time (Fig. 1B, lower panels). These results indicate that fungal viability is required for the association, perhaps due to active release of molecules such as nutrients from the fungus. However, it is possible that heat treatment causes modifications of fungal cell wall components that result in weak attachment of *S.* Typhimurium to dead mycelia. The rapidity and duration of the attachment suggest that the *S.* Typhimurium–*A. niger* interaction is causal and stable.

### *S*. Typhimurium is attracted towards *A. niger*

**Fig 2:**
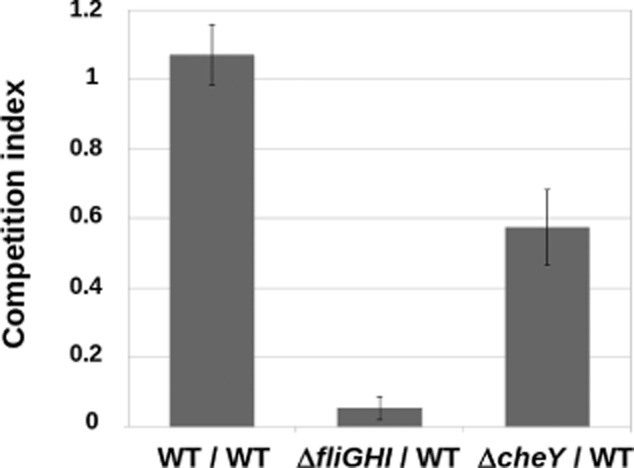
Ratios of attached bacteria normalized to input ratios. Mixtures of mutant : wild-type *S*. Typhimurium strains or wild type with an antibiotic resistance gene : wild type with no resistance marker (WT : WT, as a control) at 1:1 proportion were incubated with *A. niger* mycelia for 1 h. Mycelia were then rinsed and attached bacteria were detached and plated onto selective media. The ratio of attached bacteria was calculated for each mutant and a control wild type, and subsequently normalized to the corresponding ratios of the input mixtures. Error bars represent standard deviation of the values obtained in three independent biological replicates (*n* = 3).

Because *S.* Typhimurium is found associated with the fungal hyphae within minutes of co-inoculation, we hypothesized that the bacteria might be using directed motility and chemotaxis (Krell *et al*., 2011) to move towards the fungus. To test if motility and chemotaxis were involved in the initial attraction of bacteria to the fungus, we performed competition experiments where we analysed attachment of wild-type bacteria compared with either a motility mutant that completely lacks flagella (*fliGHI*) or a mutant that can swim, but is defective in chemotaxis (*cheY*). Competition between two differentially labelled wild-type strains was used as a control. When wild-type cells were challenged against each other the competition index was 1, which is what would be expected if equivalent numbers of each strain attached to the fungi (Fig. 2). The ability to swim was essential for fungal colonization by *S.* Typhimurium as the mutant without flagella was decreased to only 5% of the attached population when competed with the wild type. The chemotaxis mutant also showed a defect, although not as pronounced; the *cheY* mutant showed a 40% reduction in the attachment to the fungus with respect to the wild type (Fig. 2). These results indicate that *S.* Typhimurium must be able to swim directionally towards the fungus in order for colonization to occur and the bacterial cells are able to sense the fungus in order to actively move towards it. All in all these results suggest that the interaction is specific.

**Fig 3:**
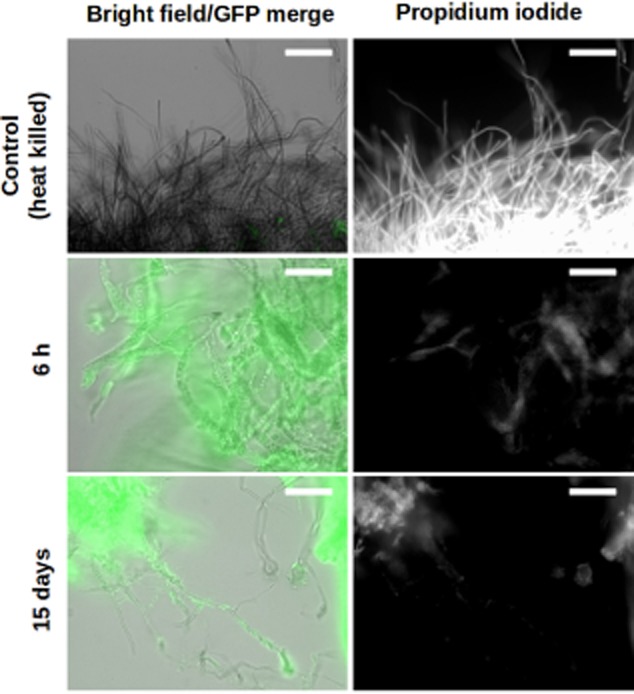
*Aspergillus niger* remains alive when colonized by *S*. Typhimurium. Epifluorescence microscopy overlay images of live and heat-killed *A. niger* mycelia (filaments, grey) co-incubated with sfGFP-labelled *S*. Typhimurium cells (false coloured green, left panels) stained with propidium iodide (white, right panels). Scale bars: 50 μm.

**Fig 4:**
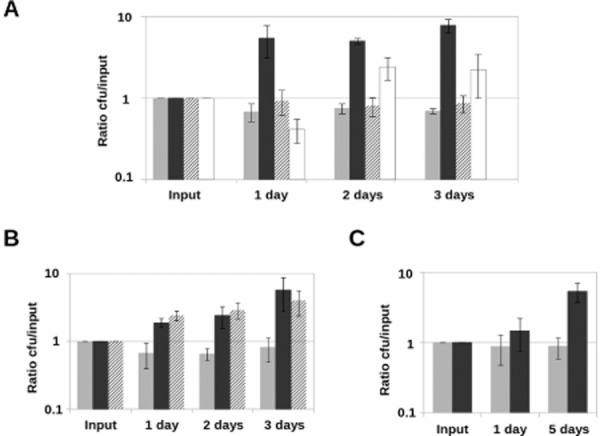
Bacterial growth requires live fungi or dead fungal lysate. Bacteria were detached and quantified at different time points, and cfu were normalized with respect to input values.A. Light grey bars represent growth of bacteria alone, dark grey bars represent growth of *S*. Typhimurium in co-incubation with live *A. niger*, striped bars represent bacterial growth in co-incubation with heat-killed fungal filaments (washed after killing) and white bars represent growth in co-incubation with live fungi separated by a semipermeable membrane.B. Light grey bars represent growth of bacteria alone, dark grey bars represent growth of *S*. Typhimurium in co-incubation with live *A. niger* and striped bars represent bacterial growth in co-incubation with heat-killed fungi in the same potassium phosphate buffer where it was killed.C. Light grey bars represent growth of bacteria alone and dark grey ones represent growth of *S*. Typhimurium in presence of *A. niger* filtrate. All co-incubations were performed in potassium phosphate buffer. Error bars represent standard deviation of the values obtained in three independent biological replicates (*n* = 3). *Y*-axis values are represented in logarithmic scale.

**Fig 5:**
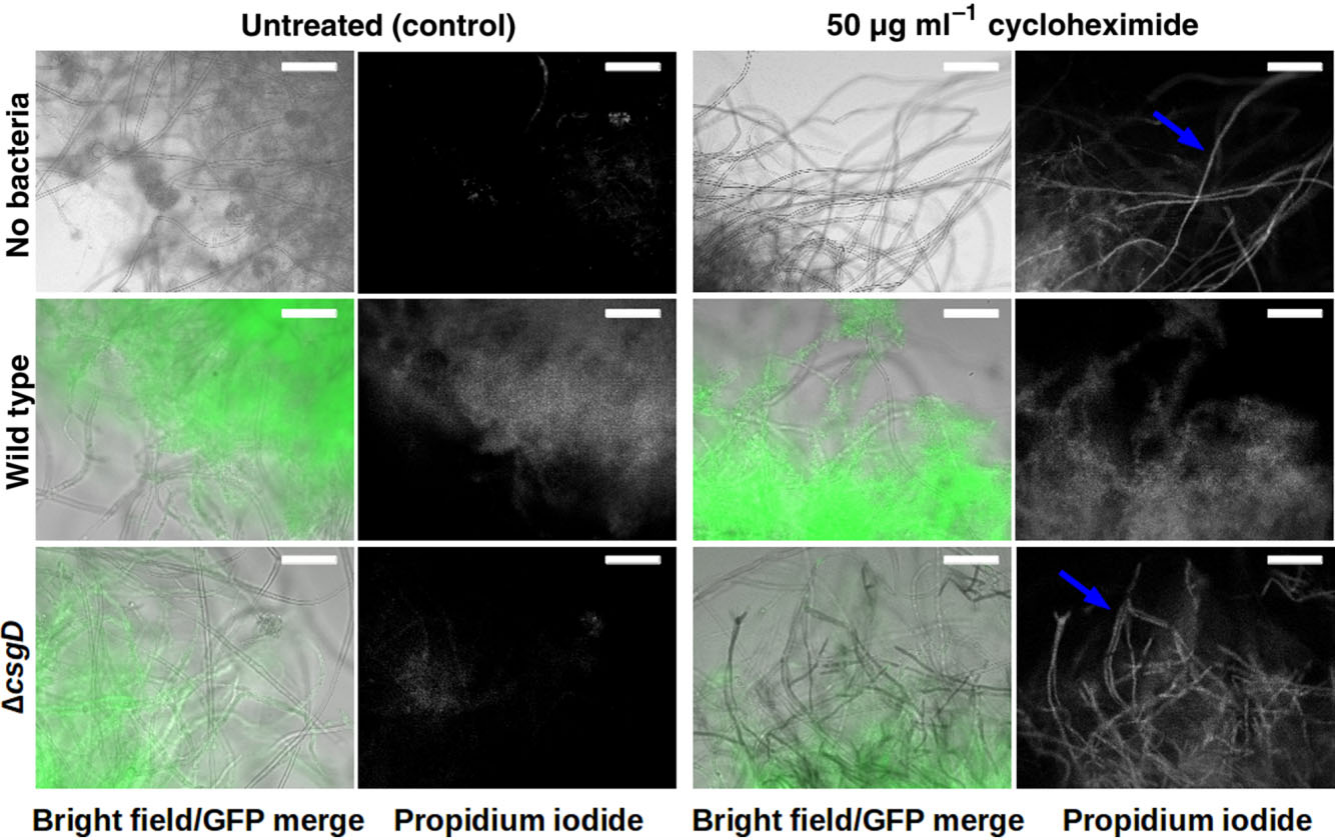
Bacteria protect *A. niger* from killing by cycloheximide. Epifluorescence microscopy overlay images of *A. niger* mycelia (filaments, grey) grown alone or co-incubated with wild-type or *ΔcsgD* mutant *S*. Typhimurium cells tagged with sfGFP (false coloured green) for 48 h. Samples were either untreated or exposed to cycloheximide for 12 h prior to staining with propidium iodide (white). Scale bars: 50 μm.

### Chitin does not function as a signal or as a source of energy in the interaction

Recently, chitin was found to be essential for bacterial attachment to *A. niger* hyphae (Brandl *et al*., 2011). We hypothesized that chitin may also participate in regulatory mechanisms involved in the interaction or, alternatively, that it might be utilized by bacteria as a source of energy during the association. To test this, a set of *S.* Typhimurium mutants involved in chitin uptake, degradation or catabolism (see Fig. S1) were tested for their ability to interact with the fungus. The mutants were not affected in the interaction (Fig. S1), suggesting that chitin does not act as a signal or as an important source of energy in the interaction between *S.* Typhimurium and *A. niger*.

### The two main *Salmonella* pathogenicity islands do not participate in the interaction

*Salmonella* Typhimurium possesses several genomic islands called *Salmonella* pathogenicity islands (SPIs), where many virulence factors are encoded. The main two SPIs are the so-called SPI-1 and SPI-2 and each one encodes a type III secretion system (T3SS). T3SSs are involved in the translocation of virulence effectors into the cytoplasm of host cells (Fàbrega and Vila, 2013). In order to test whether protein translocation or SPIs are involved in the *S.* Typhimurium–*A. niger* interaction, strains harbouring deletions of either SPI-1, SPI-2 or both were tested for their ability to interact with the fungus. None of the single mutants or the double mutant showed differences with the wild type (Fig. S2A), indicating that SPI-1 and SPI-2 do not participate in the association.

### Mutants in attachment factors are not defective for the interaction

Brandl and colleagues (2011) discarded the participation of the fimbrial operons *bcf*, *fim*, *lpf*, *pef*, *stf*, *std*, *stb*, *sth* and *stc* in the *S.* Typhimurium–*A. niger* interaction. To investigate if other known attachment factors might be important for the interaction with the fungus, several mutants were constructed. The fimbrial operons *sti* and *stj*, the putative fimbrial operon *sadAB*, and the adhesins *misL* (located in SPI-3), *shdA* (located in CS54 island), *siiABCDEF* (located in SPI-4) and *bapABCD* (located in SPI-9) were deleted and tested individually. None of the individual mutants showed defects in the interaction (Fig. S2B), suggesting that none of these attachment factors are required for the association with the fungus. Alternatively, functional redundancy could have masked any effects from single mutants. The study of the effects of double, triple or multiple mutants might help clarify this issue.

### The interaction does not cause any noticeable negative effect on either of the participants

Fungi and bacteria are able to harm each other (Wargo and Hogan, 2006). Therefore, it could be possible that the interaction between *S.* Typhimurium and *A. niger* would result in fungal or bacterial death. To investigate if fungi or bacteria were killed during the co-incubation, mycelia were co-cultured with sfGFP-expressing bacteria, retrieved at different time points, stained with PI and analysed by fluorescence microscopy (Fig. 3). As a control, heat-killed mycelia were stained and analysed. Average fluorescence intensity for the PI staining was quantified and the heat-killed control samples had an integrated density value of 1337.20 ± 239.11. In contrast, dead fungal hyphae were not observed in the co-incubation samples at any time (integrated density values from PI staining were 163.47 ± 8.77 at 6 h and 437.64 ± 16.67 at 15 days), demonstrating that the interaction does not harm the fungus. A faint and disperse PI staining can be observed in areas of high bacterial aggregation (Fig. 3). This weak signal is not specifically localized to filaments as one observes for PI staining of dead fungi. Such diffuse staining is likely due to the presence of some dead bacteria or to non-specific staining of the extracellular matrix of bacterial biofilms. Thus, we tested survival of bacteria in co-culture.

### The presence of the fungus promotes bacterial growth

In order to test bacterial survival and growth in co-culture with the fungus, *S.* Typhimurium was incubated in potassium phosphate buffer in three conditions: alone, in the presence of live *A. niger* mycelia or in the presence of heat-killed fungus that had been washed in buffer. At time zero and every 24 h for 3 days, bacterial cfu were calculated. As might be expected, potassium phosphate buffer does not support the growth of *S.* Typhimurium alone (Fig. 4A, light grey bars). However, bacterial growth was observed in the presence of live fungus (Fig. 4A, dark grey bars). In contrast, bacteria did not grow when co-cultured with heat-killed mycelia (Fig. 4A, striped bars). This suggests that bacteria can utilize metabolites produced by the fungus but are not feeding directly on fungal cells. To investigate whether attachment to the fungus is necessary for bacterial growth, the co-culture was performed in a millicell vessel where the bacteria were physically separated from the live fungus by a permeable barrier with a 0.2 μm pore size that allows for diffusion of small molecules, but not fungi or bacteria. In this case, bacterial growth was observed in the presence of live mycelia, even with a barrier (Fig. 4A, white bars). Notably, when the microbes are physically separated, the growth rate is lower than when they are co-incubated (compare Fig. 4A, white bars to Fig. 4A, dark grey bars). Thus, the attachment is not essential for growth promotion but it improves it, probably due to better diffusion and/or higher local concentration of the fungal nutrients. Because secretions from fungi were sufficient to support bacterial growth when fungi and bacteria are separated by a membrane, we revisited the experiment where dead fungi were used as a host. We reasoned that perhaps by washing the heat-killed fungal mycelia described in Fig. 4A we were removing metabolites that had been secreted in the medium or released by cells during heating. Therefore, the experiment was repeated using dead mycelia in the very same potassium phosphate buffer where the fungus was heat killed so the nutrients released by fungal lysis remained in the buffer upon co-incubation. In these conditions, the bacteria showed a similar growth rate to that observed in the presence of live fungus (Fig. 4B). This verifies that the nutrients used by *S.* Typhimurium are fungal metabolites and, importantly, indicates that these fungal nutrients are not produced as a consequence of the interaction but were already being synthesized by the fungus before the introduction of bacteria. We next wondered if using fungal filtrate from a mature culture (obtained after 3 days of fungal growth) would support the growth of *S.* Typhimurium. At 24 h, bacterial growth in the presence of 3-day-old fungal filtrate was poor but after 5 days it was comparable with that of cells grown in the presence of the fungus (compare Fig. 4C with Fig. 4B). This suggests that the utilization of fungal compounds by *S.* Typhimurium occurs at a relatively slow rate, but that there are sufficient nutrients present to support growth even in the absence of fungal cells.

### *S*. Typhimurium biofilms protect *A. niger* against the action of cycloheximide

The results presented thus far indicate that fungal cells remain alive and that the presence of the fungus is required to stimulate bacterial growth under our co-culture conditions. We next examined if there was a benefit to the fungi. Biofilm-associated bacteria have an increased resistance to antimicrobials, starvation, desiccation and other stresses (Nickel *et al*., 1985a,b; Anriany *et al*., 2001; Scher *et al*., 2005; Lapidot *et al*., 2006; Wong *et al*., 2010). We hypothesized that *S.* Typhimurium biofilms might confer protection to *A. niger*. To test this, mycelia were incubated alone or co-incubated with either wild-type bacteria or a Δ*csgD* mutant, which is unable to form biofilms or persist on fungal filaments (Römling *et al*., 1998; Brandl *et al*., 2011). After 48 h of incubation, the antifungal agent cycloheximide was added to each culture at a final concentration of 50 μg ml^−1^. Cycloheximide kills fungi but is harmless for bacteria (Whiffen, 1948). After 12 h of exposure to cycloheximide, mycelia were stained with PI and observed by fluorescence microscopy (Fig. 5). Mycelia incubated in the absence of bacteria show extensive damage as the filaments clearly stained with PI (integrated density value: 541.89 ± 74.11). Interestingly, mycelia co-incubated with wild-type bacteria do not show any PI-staining hyphae (integrated density value: 266.12 ± 38.49), although there was diffuse PI staining around the bacterial aggregates similar to what was observed in Fig. 3. In contrast, fungi incubated in the presence of the Δ*csgD* mutant were severely affected by cycloheximide as there was distinct staining of filaments by PI (integrated density value: 550.90 ± 17.76) compared with mycelia co-incubated with wild-type bacteria (see above) or untreated controls (integrated density value of untreated mycelia alone was 215.97 ± 53.24; untreated mycelia co-incubated with wild-type bacteria was 277.78 ± 9.01 and untreated mycelia co-incubated with the Δ*csgD* mutant was 299.15 ± 22.05) (Fig. 5). Thus, *S.* Typhimurium biofilm formation protects the fungus from the toxic effects of cycloheximide.

Given that the fungus promotes bacterial growth (Fig. 4) and bacterial biofilms protect the fungus against the action of an antifungal (Fig. 5), we concluded that the interaction between *S.* Typhimurium and *A. niger* is mutualistic.

### *S*. Typhimurium and *A. niger* co-colonize maize roots

**Fig 6:**
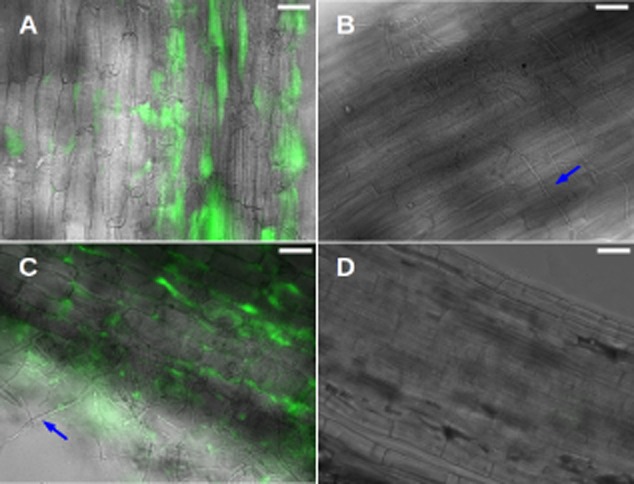
Maize roots are colonized by *S*. Typhimurium and *A. niger*. Images are overlay of transmitted light (grey) with sfGFP fluorescence (false coloured green).A. Epidermal maize root tissue colonized by sfGFP-labelled *S*. Typhimurium.B. Epidermal maize root tissue colonized by *A. niger* (blue arrow).C. Epidermal maize root tissue colonized by sfGFP-labelled *S*. Typhimurium and *A. niger*. Blue arrow points at a representative fungal filament.D. Non-inoculated maize root tissue is shown as control. Scale bars: 50 μm.

Because both *S.* Typhimurium and *A. niger* can often be found associated with plants (Perrone *et al*., 2007; Schikora *et al*., 2008), plant roots may be an environmental niche where this fungal–bacterial interaction could take place. To study that possibility, we developed a tripartite system involving the bacterium, the fungus and a plant. For these studies, we used maize (*Zea mays*) because both microorganisms have been reported to colonize this plant (Singh *et al*., 2004; Palencia *et al*., 2010). We used a gnotobiotic system where sterile maize roots were inoculated with either 10 mM potassium phosphate buffer (pH 7), *S.* Typhimurium, *A. niger* or co-inoculated with both microbes. At 14 days post-inoculation (dpi), plants were analysed for the presence of the organisms on the roots. *Salmonella* Typhimurium colonized maize roots in this gnotobiotic system (Fig. 6A). *Aspergillus niger* was also observed associated with the roots (blue arrow, Fig. 6B). In addition, co-colonization was observed when the fungi and bacteria were both introduced to the plant (Fig. 6C). As a negative control, an uninoculated root is shown in Fig. 6D. We next wondered if the fungal–bacterial co-colonization might have a different effect on the plant than colonization by either organism alone.

**Fig 7:**
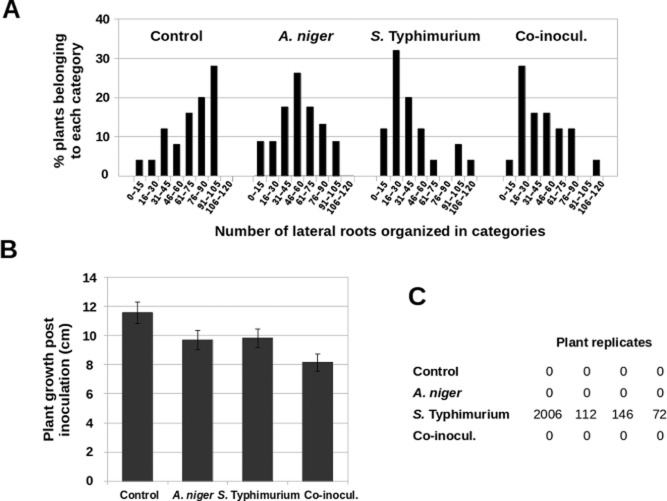
Effects of colonization by *S*. Typhimurium and *A. niger* on maize plants.A. Frequency of plants distributed in categories according to their number of lateral roots. The higher percentages correspond to high numbers of lateral roots in the distribution of control plants. In contrast, distributions of plants inoculated with bacteria, fungi or co-inoculated with both present higher percentages of plants in categories corresponding to low number of lateral roots. Ninety-two plants were analysed for this experiment.B. Growth of plants according to their height in centimetres at 14 dpi relative to 0 dpi. Fifty plants were analysed for each condition. Bars represent average values of each distribution and error bars are standard error of the mean.C. Bacterial presence in maize flag leaves (expressed in cfu g^−1^ tissue). Control plants and those inoculated with *A. niger* show no bacteria. Plants inoculated with *S*. Typhimurium show variable bacterial loads. In contrast, plants co-inoculated with bacteria and fungi do not present any bacteria in the flag leaf tissues. Sixteen plants were analysed for this experiment.

We noticed that colonization of maize roots by either *S.* Typhimurium, *A. niger* or a mixture of both microbes caused a decrease in the number of lateral roots (Lynch, 2006) and we decided to quantify this effect. To test this, groups of 23 plants treated with either buffer, bacteria, fungi or a mixture of bacteria and fungi were assayed for the number of lateral roots 14 days post-inoculation. The percentage of plants belonging to different categories according to the number of lateral roots was calculated for each inoculation, and each distribution was compared with that of the control group (plants treated with buffer alone). Plants colonized by either *S.* Typhimurium or *A. niger* individually and those co-colonized by both microbes showed a statistically significant (*P*-values below 0.01) reduction in their number of lateral roots with respect to control plants. (Fig. 7A). However, no significant difference was found between plants co-colonized by both microorganisms relative to those colonized by bacteria or fungi individually (Fig. 7A). This indicates that root development is affected by the presence of either *S.* Typhimurium or *A. niger*, but co-colonization does not alter this effect.

We also measured the effects of colonization on plant growth, as measured by plant height (from the seed to the tip of the flag leaf) (Peiffer *et al*., 2014). To do this, the increase in plant height at 14 dpi relative to the height of the plant at 0 dpi was assessed for groups of 50 plants treated with buffer, bacteria, fungi or a mixture of both. The distributions of data were compared using one-way ANOVA test (*P*-value < 0.01) and resulted to be statistically different. We observed that individual colonization by either bacteria or fungus caused a minor decrease in plant height (Fig. 7B). However, the effect of co-colonization is greater (Fig. 7B). This suggests additive or synergistic effect of fungal–bacterial co-colonization on suppression of maize growth.

Finally, it has been reported that *S.* Typhimurium is able to invade and survive inside many plants (Jablasone *et al*., 2005; Schikora *et al*., 2008; Gu *et al*., 2011; 2013; Ge *et al*., 2013). We thus sought to determine if *S.* Typhimurium is also able to invade maize plants and, if so, whether invasion is affected by the interaction with *A. niger*. To this end, roots of groups of four plants were inoculated with either buffer, bacteria, fungi or both. At 14 dpi, the 1 cm at the tip of the flag leaf of each plant was cut and assessed for the presence of bacteria. Leaf tissue was weighed, homogenized and cfu g^−1^ tissue was calculated for each plant. As expected, control plants and plants inoculated with *A. niger* alone showed no bacteria (Fig. 7C). In contrast, all plants inoculated only with *S.* Typhimurium consistently showed the presence of bacteria in leaf tissue, although the total bacterial counts for each plant varied, as was previously observed in tomato plants (Gu *et al*., 2013). Surprisingly, bacteria were never detected in leaf samples from plants co-colonized by both bacteria and fungi (Fig. 7C). Given that the levels of colonization of maize roots by *S.* Typhimurium are equivalent in the presence or the absence of *A. niger* (Fig. S3), it seems that fungi are able to affect bacterial ability to invade plants.

This study presents evidence of a fast-forming, stable and specific interaction between *S.* Typhimurium and *A. niger*. This association promotes bacterial growth and results in fungal protection by bacterial biofilms, indicating the mutualistic nature of the relationship. Moreover, the interaction takes place in maize roots and colonization by either organism alone causes a slight decrease in plant growth. However, co-colonization has a greater effect. This work also unveiled that *S.* Typhimurium is able to invade maize, as has been previously found for other plants (Gu *et al*., 2011). However, co-colonization with *A. niger* inhibits the invasion of maize by *S.* Typhimurium.

## Supporting information

**Fig. S1.** Epifluorescence microscopy overlay images of mycelia of *A. niger* co-incubated with wild type and different mutant strains of *S.* Typhimurium tagged with sfGFP (false coloured green). A. Mutants lacking the proteins involved in chitooligosaccharide uptake ChiP and ChiQ (Figueroa-Bossi *et al*., 2009) or ChiA, which is the only potential chitinase encoded in the *S.* Typhimurium genome that has been shown to have activity *in vitro* (Larsen *et al*., 2011), show extensive attachment to the fungus at both early incubation time and after 24 h. B. The Δ*chiX* mutant, which overexpresses *chiPQ* (Figueroa-Bossi *et al*., 2009), does not show any difference with respect to the wild type regarding interaction with the fungus after 6 h of co-incubation. C. At 4 h of co-incubation, the mutants involved in chitobiose transport and catabolism Δ*chbC* and Δ*chbF* (Keyhani and Roseman, 1997) show similar levels of interaction with the fungus than the wild type. Scale bars: 50 μm. **Fig. S2.** Epifluorescence microscopy overlay images of mycelia of *A. niger* co-incubated with wild-type and different mutant strains of *S.* Typhimurium tagged with sfGFP (false coloured green). A. None of the single mutants Δ*SPI-1* and Δ*SPI-2* nor the double mutant Δ*SPI-1* Δ*SPI-2* show any difference with respect to the wild type in terms of attachment to the fungus at early incubation time (3 h). B. Mutants Δ*sti*, Δ*shd*, Δ*misL*, Δ*bap*, Δ*stj*, Δ*sad* and Δ*sti* show similar attachment than the wild type at 4 h of co-incubation. Scale bars: 50 μm. **Fig. S3.** Epifluorescence dissecting microscopy images of maize root colonization by sfGFP-labelled *S.* Typhimurium alone or in co-colonization with non-labelled *A. niger*. Bacterial attachment takes place at equivalent levels when *S.* Typhimurium is alone (top panels) and when it is in co-colonization with *A. niger* (bottom panels). Scale bars: 1 mm. **Table S1.** Relevant strains used in this work. **Table S2.** DNA oligonucleotides used in this work.
