# Supplementary material for: Mutualistic interaction between Salmonella enterica and Aspergillus niger and its effects on Zea mays colonization
Source: Microb Biotechnol. 2014 Oct 29;7(6):589–600. doi: 10.1111/1751-7915.12182 (PMC4265077; doi:10.1111/1751-7915.12182)
Supplement: Supplementary file 1 [file mbt20007-0589-sd1.doc]

**Mutualistic interation between *Salmonella enterica* and *Aspergillus niger* and its effects on *Zea mays* colonization**

Roberto Balbontín*, Hera Vlamakis and Roberto Kolter

Department of Microbiology and Immunobiology, Harvard Medical School, Boston, Massachusetts, USA.

Running title: *Salmonella-Aspergillus* interaction on maize

*E-mail: roberto_balbontin@hms.harvard.edu

**SUPPORTING INFORMATION**


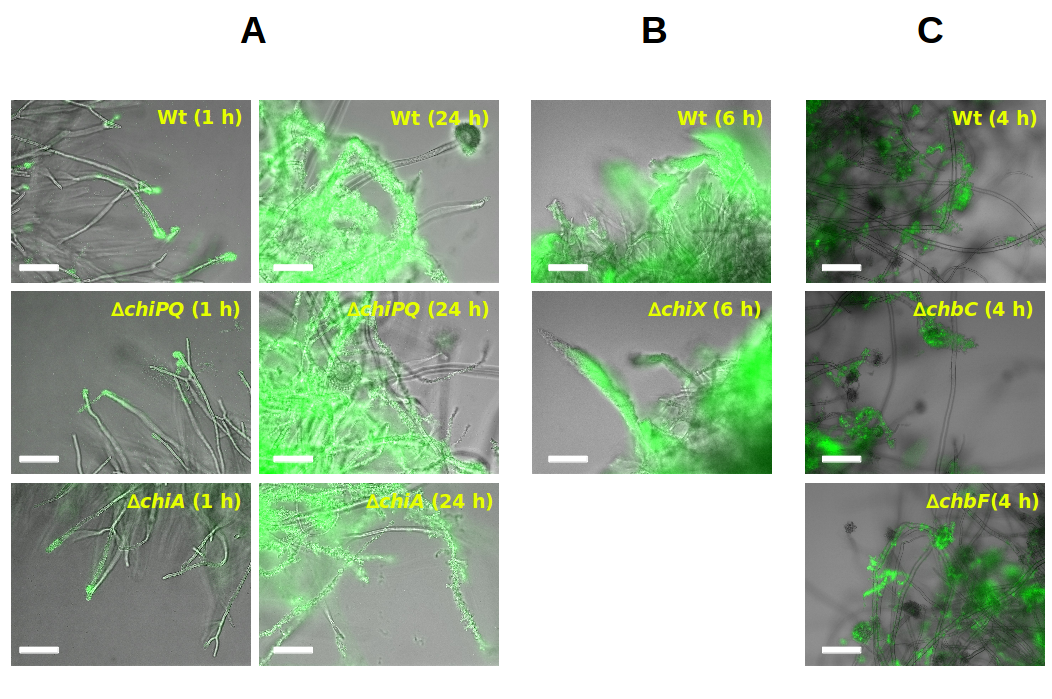
**Figure S1.** Epifluorescence microscopy overlay images of mycelia of *A. niger* co-incubated with wild type and different mutant strains of *S.* Typhimurium tagged with sfGFP (false colored green). **A.** Mutants lacking the proteins involved in chitooligosaccharide uptake ChiP and ChiQ (Figueroa-Bossi *et al.*, 2009) or ChiA, which is the only potential chitinase encoded in the *S.* Typhimurium genome that has been shown to have activity *in vitro* (Larsen *et al.*, 2011) show extensive attachment to the fungus at both early incubation time and after 24 hours. **B.** The *ΔchiX* mutant, that overexpresses *chiPQ* (Figueroa-Bossi *et al.*, 2009), does not show any difference with respect to the wild type regarding interaction with the fungus after 6 hours of co-incubation. **C.** At 4 hours of co-incubation, the mutants involved in chitobiose transport and catabolism *ΔchbC* and *ΔchbF* (Keyhani and Roseman, 1997) show similar levels of interaction with the fungus than the wild type. Scale bars: 50 μm.


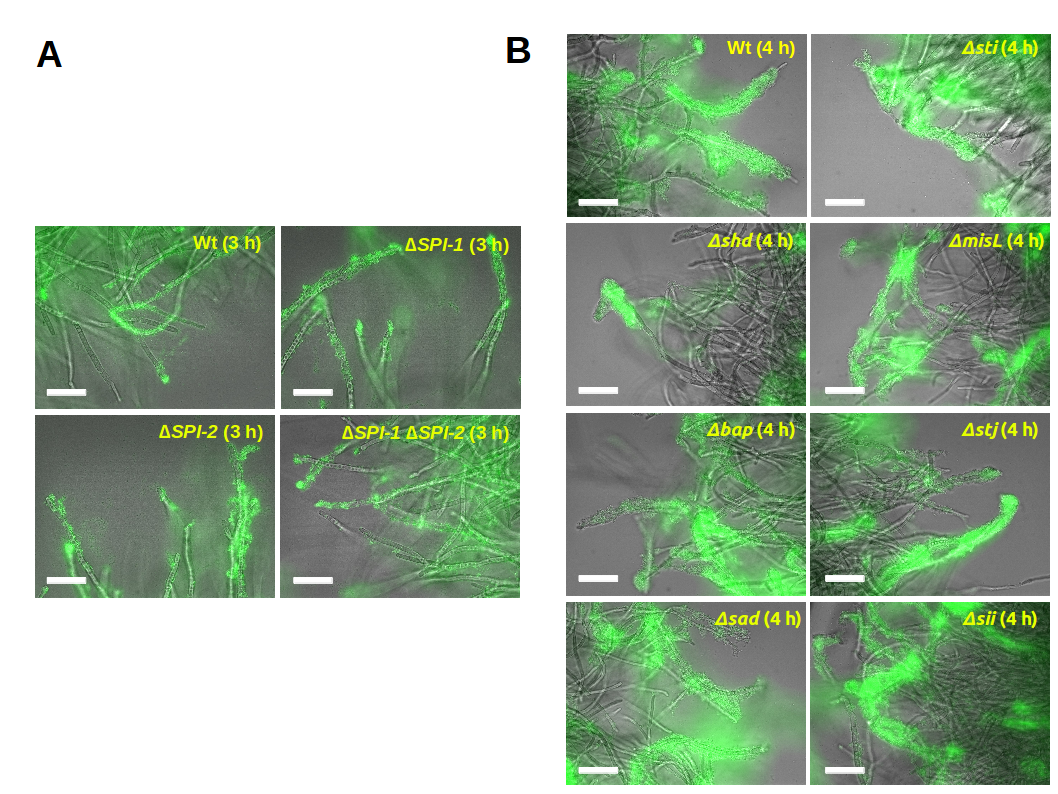


**Figure S2.** Epifluorescence microscopy overlay images of mycelia of *A. niger* co-incubated with wild-type and different mutant strains of *S.* Typhimurium tagged with sfGFP (false colored green). **A.** None of the single mutants *ΔSPI-1* and *ΔSPI-2* nor the double mutant *ΔSPI-1 ΔSPI-2* show any difference with respect to the wild type in terms of attachment to the fungus at early incubation time (3 hours). **B.** Mutants *Δsti*, *Δshd*, *ΔmisL*, *Δbap*, *Δstj*, *Δsad* and *Δsti* show similar attachment than the wild type at 4 hours of co-incubation. Scale bars: 50 μm.


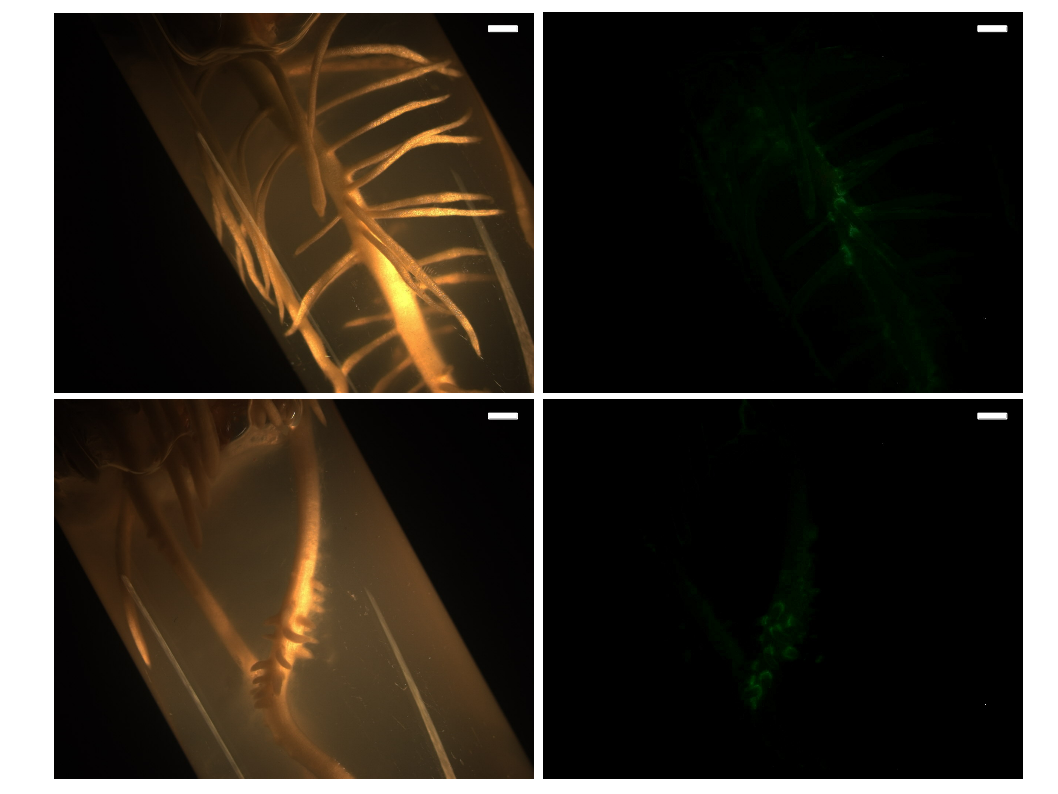


**Figure S3.** Epifluorescence dissecting microscopy images of maize root colonization by sfGFP-labeled *S.* Typhimurium alone or in co-colonization with non-labeled *A. niger*. Bacterial attachment takes place at equivalent levels when *S.* Typhimurium is alone (top panels) and when it is in co-colonization wth *A. niger* (bottom panels). Scale bars: 1 mm.

**Table S1.** Relevant strains used in this work.

**Strain^a^  Organism Relevant genotype Source or reference**

ZK3055^b^ *A. niger* wild type H.A. Burge

ZK2851 *S.* Typhimurium wild type M. Hensel

RB164 *S.* Typhimurium *P_constitutive_sfGFP* this work

RB176 *S.* Typhimurium *∆chiPQ, P_constitutive_sfGFP* this work

RB183 *S.* Typhimurium *∆chiA, P_constitutive_sfGFP* this work

RB206 *S.* Typhimurium *∆cheY* this work

RB207 *S.* Typhimurium *∆fliGHI* this work

RB225 *S.* Typhimurium *∆chbC, P_constitutive_sfGFP* this work

RB226 *S.* Typhimurium *∆chbF, P_constitutive_sfGFP* this work

RB229 *S.* Typhimurium *∆[chiX-ybaP], P_constitutive_sfGFP* this work

RB230 *S.* Typhimurium *∆csgD, P_constitutive_sfGFP* this work

RB231 *S.* Typhimurium STM14_4239 ::*tetAR* this work

RB242 *S.* Typhimurium *∆[avrA-invH], P_constitutive_sfGFP* this work

RB243 *S.* Typhimurium *∆[ssaU-ssaB]::scar, P_constitutive_sfGFP* this work

RB244 *S.* Typhimurium *∆[avrA-invH], ∆[ssaU-ssaB], P_constitutive_sfGFP* this work

RB258 *S.* Typhimurium *∆[bapA-bapD], P_constitutive_sfGFP* this work

RB259 *S.* Typhimurium *∆[STM14_3086-shdA], P_constitutive_sfGFP* this work

RB260 *S.* Typhimurium *∆misL, P_constitutive_sfGFP* this work

RB261 *S.* Typhimurium *∆[sadA-sadB], P_constitutive_sfGFP* this work

RB263 *S.* Typhimurium *∆[siiA-siiF], P_constitutive_sfGFP* this work

RB264 *S.* Typhimurium *∆[stiA-stiH], P_constitutive_sfGFP* this work

RB265 *S.* Typhimurium *∆[STM14_5495-STM14_5491], P_constitutive_sfGFP* this work

^a^ All strains are derived from *Salmonella enterica* serovar Typhimurium strain ZK2851, except  ^b^.

**Table S2.** DNA oligonucleotides used in this work.

**Name Target Sequence (5'->3')**

ORB002 RB164-2-rev aagccatggtacctttctcctctttaatgaattcggtcagtgcgtcctgctgatgtgctc

ORB003 RB164-3-fwd ctgaccgaattcattaaagaggagaaaggtaccatggcttctaaaggtgaagaactgttc

ORB007 RB164-1-fwd tcggcttcgccaaaacgatcggcgacataatcccactacgcctagtgcttggattctcac

ORB008 RB164-4-rev gatatgtcgtttgtcgatgtccaggcattgaaggacaatcgatgcctctagatttaaatgctcg

ORB010 malX-Ext-fwd ggtggcaaaatccatatccg

ORB011 malY-Ext-rev ggaaaacatcgtgagtctgg

ORB024 chiP-Tet-fwd tgtcggcagcaatttatacgtcaaagaggattaacttatgttaagacccactttcacatt

ORB027 Tet-chiQ-rev gcggaaaacattcgcccgtgggtggcaaggcagggttttactaagcacttgtctcctg

ORB028 chiP-Ext-fwd gtcggcagcaatttatacgtc

ORB029 chiQ-Ext-rev aaaacattcgcccgtgggtg

ORB043 chiA-Tet-fwd gctgcgacaattttgaaacgtaaaaggaaatttgaaaatgttaagacccactttcacatt

ORB044 Tet-chiA-rev aatcatgaagcccaatacatcggcttaataccgtgtattactaagcacttgtctcctg

ORB045 chiA-Ext-fwd gcctgtaacgattatggacc

ORB046 chiA-Ext-rev ctactcttcctctgtccc

ORB057 cheY-Tet-fwd gaaccaggagtagtattttatggcggataaagagcttaaattaagacccactttcacatt

ORB058 Tet-cheY-rev ttgcatcatcatcgcatcctcacatgcccagtttctcaaactaagcacttgtctcctg

ORB059 cheY-Ext-fwd ggagtagtattttatggcgg

ORB060 cheY-Ext-rev gttgcatcatcatcgcatcc

ORB061 fliG-Tet-fwd gtggcgctggtcattcgccagtggatgagtaacgatcatgttaagacccactttcacatt

ORB062 Tet-fliI-rev cgccatgttgtgccatgatcgtcgccctcctgctttatcactaagcacttgtctcctg

ORB063 fliG-Ext-fwd gcgtattcgcgaaatgtcag

ORB064 fliI-Ext-rev gtcgccctcctgctttatc

ORB079 csgD-Tet-fwd cagctgtcagatgtgcgattaaaaaaagtggagtttcatcttaagacccactttcacatt

ORB080 Tet-csgD-rev ctctgctgctacaatccaggtcagatagcgtttcatggccctaagcacttgtctcctg

ORB081 csgD-Ext-fwd catctgtcagtacttctggtg

ORB082 csgD-EXt-rev gtttccggtagcgaacag

ORB083 STM14_4239-Tet-fwd atcatcccggctatgaatttgtcgcgcaacgctgaaggcactaagcacttgtctcctg

ORB084 Tet-rtcB-rev ctgcgtctggccatacaaggtgtagatccaccagcatttgttaagacccactttcacatt

ORB085 STM14_4239-Ext-fwd gattacatctttcccgatgc

ORB086 rtcB-Ext-rev cattagggtggcgacaag

ORB098 bapA-P1-fwd gttaattaatacacagagcaaatccatcaggagctgatttgtgtaggctggagctgcttc

ORB099 P2-bapD-rev cacgcgtgaccagcccccgtatcttcttatcttcaacgatcatatgaatatcctccttag

ORB100 bapA-Ext-fwd tacgaaagcactagtcaggc

ORB101 BapD-Ext-rev caccttgggtttcgcaaatc

ORB102 CS54-P1-fwd ttttcatagaggcgtttttatttaacgacagggatatcatgtgtaggctggagctgcttc

ORB103 P2-CS54-rev tcaaaaccgggcagggaacacccgcccggttttgtctaaccatatgaatatcctccttag

ORB104 CS54-Ext-fwd ggcctgttagttatgattcc

ORB105 CS54-Ext-rev ggtgtagccataatctgacg

ORB106 misL-P1-fwd cgatgtggaagacgctttacgccataatgcaggaggcagagtgtaggctggagctgcttc

ORB107 P2-misL-rev taaaagccgctgaagatcagcggctctgttgttacctgaacatatgaatatcctccttag

ORB108 misL-Ext-fwd cgatgtggaagacgctttac

ORB109 misL-Ext-rev gttcatgaaacctatcagcc

ORB110 sadA-P1-fwd actatttatattatataaattattaacaaggatatttcatgtgtaggctggagctgcttc

ORB111 P2-sadB-rev ttatgccattgcctttgatgtcgggagtgttgttacttcacatatgaatatcctccttag

ORB112 sadA-Ext-fwd gcaatactcgctgaatgatg

ORB113 sadB-Ext-rev gtcctgccaatattgttgtg

**Table S2.** DNA oligonucleotides used in this work (continued).

**Name Target Sequence (5'->3')**

ORB118 siiA-P1-fwd acaaaaacattttattcacaatgtaatatcaggagacaacgtgtaggctggagctgcttc

ORB119 P2-siiF-rev aagcagtaccacctgataacagcgacaagcgctgcttattcatatgaatatcctccttag

ORB120 siiA-Ext-fwd gtgggcgcataaaaatggcg

ORB121 siiF-Ext-rev cttattgaagacgttgccgac

ORB122 stiA-P1-fwd actatgtcaatatttataatatcttaaaggatcaaagatagtgtaggctggagctgcttc

ORB123 P2-stiH-rev cgggtcggtaggtgaggcttgtcgaaacccaacgcttctccatatgaatatcctccttag

ORB124 stiA-Ext-fwd cgccgtaaaaatcatcggac

ORB125 stiH-Ext-rev gttgcgcttcgcttcacttc

ORB126 STM14_5495-P1-fwd catgtcatttggaaatatacttaatcgttagggattactggtgtaggctggagctgcttc

ORB127 P2-stjB-rev taagattgtgaagggggccagagcaggcccccttcatcgtcatatgaatatcctccttag

ORB128 STM14_5495-Ext-fwd cgcgcattacatgtcatttg

ORB129 stjB-Ext-rev gtactgctgggcgataaag
